# Environmental Barcoding Reveals Massive Dinoflagellate Diversity in Marine Environments

**DOI:** 10.1371/journal.pone.0013991

**Published:** 2010-11-15

**Authors:** Rowena F. Stern, Ales Horak, Rose L. Andrew, Mary-Alice Coffroth, Robert A. Andersen, Frithjof C. Küpper, Ian Jameson, Mona Hoppenrath, Benoît Véron, Fumai Kasai, Jerry Brand, Erick R. James, Patrick J. Keeling

**Affiliations:** 1 The Biodiversity Research Centre, University of British Columbia, Vancouver, British Columbia, Canada; 2 Department of Geology, State University of New York at Buffalo, Buffalo, New York, United States of America; 3 Provasoli-Guillard National Center for Culture of Marine Phytoplankton, Bigelow Laboratory for Ocean Sciences, West Boothbay Harbor, Maine, United States of America; 4 Culture Collection of Algae and Protozoa, Scottish Association for Marine Science, Scottish Marine Institute, Oban, United Kingdom; 5 Australian National Algae Culture Collection, CSIRO Marine and Atmospheric Research, Hobart, Australia; 6 Forschungsinstitut Senckenberg, Deutsches Zentrum für Marine Biodiversitätsforschung (DZMB), Wilhelmshaven, Germany; 7 Algobank-Caen, Université de Caen Basse-Normandie, Caen, France; 8 National Institute for Environmental Studies, Tsukuba, Japan; 9 School of Biological Sciences, University of Texas at Austin, Austin, Texas, United States of America; University of Canterbury, New Zealand

## Abstract

**Background:**

Dinoflagellates are an ecologically important group of protists with important functions as primary producers, coral symbionts and in toxic red tides. Although widely studied, the natural diversity of dinoflagellates is not well known. DNA barcoding has been utilized successfully for many protist groups. We used this approach to systematically sample known “species”, as a reference to measure the natural diversity in three marine environments.

**Methodology/Principal Findings:**

In this study, we assembled a large cytochrome *c* oxidase 1 (COI) barcode database from 8 public algal culture collections plus 3 private collections worldwide resulting in 336 individual barcodes linked to specific cultures. We demonstrate that COI can identify to the species level in 15 dinoflagellate genera, generally in agreement with existing species names. Exceptions were found in species belonging to genera that were generally already known to be taxonomically challenging, such as *Alexandrium* or *Symbiodinium*. Using this barcode database as a baseline for cultured dinoflagellate diversity, we investigated the natural diversity in three diverse marine environments (Northeast Pacific, Northwest Atlantic, and Caribbean), including an evaluation of single-cell barcoding to identify uncultivated groups. From all three environments, the great majority of barcodes were not represented by any known cultured dinoflagellate, and we also observed an explosion in the diversity of genera that previously contained a modest number of known species, belonging to Kareniaceae. In total, 91.5% of non-identical environmental barcodes represent distinct species, but only 51 out of 603 unique environmental barcodes could be linked to cultured species using a conservative cut-off based on distances between cultured species.

**Conclusions/Significance:**

COI barcoding was successful in identifying species from 70% of cultured genera. When applied to environmental samples, it revealed a massive amount of natural diversity in dinoflagellates. This highlights the extent to which we underestimate microbial diversity in the environment.

## Introduction

Assessing biodiversity in the microbial world has always been a difficult problem: not only are microorganisms inherently more difficult to examine and differentiate by classical methods, but it is also not clear if the theoretical taxonomic frameworks, applied to more familiar life forms, even apply to the diversity of microbial life. Even the validity of the species concept is debatable for some protist groups. Within microbial eukaryotes, the protists, there is a persistent debate over how much diversity exists, irrespective of how we divide it up. On one side of the debate it is argued that protist diversity typically consists of a relatively few cosmopolitan species because their small size allows them to live ubiquitously [1]–[3]. The alternative argument is that the microscopic size of protists allows greater opportunity for cosmopolitan existence, but at the same time factors such as their sheer abundance, short generation time and ability to reproduce asexually allows for greater endemism [4]. At the heart of this debate are the difficulties in estimating diversity. One recent review summarizes estimates that vary from 90,000 to 300,000 protist species [4]. However, morphology can mask hidden genetic diversity and morphotypes can easily be misinterpreted [5]. Cosmopolitan genera can exist as multiple distinct genetic and even reproductive entities [6], [7]. Furthermore, many genetically divergent organisms can appear identical due to the lack of recognizable characters to distinguish them [8]–[11] and, conversely, morphologically distinct entities have also been shown to be genetically identical [11], [12]. With the ongoing decline in taxonomic expertise [13] the description of new species is also on the decline.

The application of molecular systematics to various protist lineages has revealed unexpected levels of diversity and a surge in the documentation of morphologically cryptic species [11], [14]–[20]. DNA barcoding methodology described by Hebert and colleagues [21] has already revealed novel diversity in protist taxa using the COI marker including red algae [22], [23], brown algae [24], [25], diatoms [26] and the ciliate genus *Tetrahymena* [27]. Here, we have used COI barcoding to examine the diversity of cultured and uncultured dinoflagellates.

Dinoflagellates are an ancient and evolutionarily complex group of protists, members of which occupy every major ecological niche from primary producers to parasites (reviewed in [28], [29]) and are famous for forming harmful red tides [30]–[32]. It is estimated that there are about 2,000 species of extant dinoflagellates, about half of which are photosynthetic. Marine strains are especially well represented in culture collections [28]. Because of their ecological and evolutionary importance, dinoflagellates have a relatively well developed taxonomy for certain lineages but many taxa are uncharacterized or misplaced. There is a strong descriptive bias toward species that are large with distinct morphological features (e.g. thecal plates) as well as those that are commercially important and/or cultivatable. Here we have used the large numbers of dinoflagellates available in public and private culture collections to establish a baseline of dinoflagellate DNA barcode diversity. By doing so, we can address a number of important questions. First, culture collections rely on depositors for correct species names, which can be inaccurate [33], [34]. Therefore, our baseline survey of 336 barcodes tied to specific cultures from 11 public and private culture collections allows for an accurate assessment of culture collection identifications, and provides a means to correctly identify future accessions. Secondly, by applying species diversity values from our systematic survey of characterized, cultured dinoflagellates to environmental samples, we can estimate levels of natural diversity and gauge how much natural diversity is represented in culture collections.

Recently, DNA barcoding was used to assess freshwater and brackish dinoflagellates using two mitochondrial markers, a small number of COI (cytochrome *c* oxidase 1) barcodes and a larger database of mitochondrial cytochrome *b* (cob) barcodes. This study revealed a high level of diversity in these environments including unexpected species, although diversity estimates were hampered by a limited cob database [35]. As an estimated 77% of recognized dinoflagellate species are found in marine systems [28], [36] there is potentially an even larger diversity of marine dinoflagellates from environmental studies of marine alveolates [17], [37], [38]. One example is the exclusively marine genus, *Symbiodinium,* which displays diversity levels similar to that of orders in other dinoflagellate taxa [38]. In addition, some studies show that some freshwater or brackish species are not closely related to their marine counterparts [39], [40] and one recently divergent freshwater to brackish lineage demonstrated unusually high levels of *cob* divergence [41]. Similar results have been found for other protist groups [42]–[45]. Therefore it is a concern that genetic distances may be distorted in some taxa when calculating species-level genetic distances. To maximize criteria for DNA barcode-based species identification, COI (the standard barcode marker) was used as it has substantial representation in sequence databases and can potentially be compared with other protist species. COI barcoding was comprehensively applied to previously identified culture collection strains of marine dinoflagellates. These had been largely identified morphologically and some with additional molecular markers, most commonly the small rDNA subunit (SSU), the large rDNA subunit (LSU), the internal transcribed spacer regions of rDNA (ITS), and for some *Symbiodinium* strains, a hypervariable region within Domain V of the chloroplast 23S rDNA (23S-rDNA).This was done in order to get as much accuracy as possible for our database (see for example a demonstration that the accuracy of species identification falls off in poorly characterized genera of cowries and leads to an overestimate of ‘unknown’ diversity). Another barcode marker to consider is ITS, which performed well in a study by Litiker and colleagues [46]. However generally, ITS is highly variable, with indels and paralogues which caused multiple peaks when directly sequenced (unpublished results). It would be worthwhile, however, to consider ITS as a potential marker for low-diversity genera such as *Alexandrium*.

Our survey successfully identified 101 strains (cultures with separate identification labels) from species belonging to 15 of 18 genera from culture collections to species level with a good correlation between named species and its COI sequence in most sub-groups. Nevertheless, several cases of cryptic diversity within culture collections were evident, particularly in the genus *Scrippsiella*. Variable levels of diversity in COI were observed between species belonging to different genera, also observed with the cob marker in dinoflagellates barcodes [35]. A large number of dinoflagellate strains were identified to species or in the case of *Symbiodinium* strains to the clade level. We also characterized 713 environmental barcodes from three marine environments: the Northeast Pacific, Northwest Atlantic, and Caribbean. The inferred species diversity in environmental barcodes greatly exceeded the collective diversity of all public culture collections. Indeed, from the 603 non-identical environmental barcodes only 51 sequences could be attributed to cultured species, and using the estimates of within-species diversity from the cultures 91.5% of all environmental sequences would represent unknown species. Moreover, significant expansions in several genera were seen in environmental samples: the most extreme of which were the barcodes from Northeast Pacific samples, nearly one third of which clustered with the close relatives *Karlodium* and *Karenia*, which are relatively species-poor genera. Taken together, these data suggest that we have substantially underestimated dinoflagellate diversity in the marine environment, and that our culture collections, even though they are biased towards photosynthetic, planktonic dinoflagellates, represent only a small fraction of natural diversity.

## Results

### Evaluating COI as a barcode marker for dinoflagellates

Out of 669 culture collection samples from 11 collections, we retrieved 566 COI amplicons as some taxa failed to amplify (most commonly, these were *Amphidinium* sp., *Heterocapsa* sp., *Oxyrrhis* sp. and some unknown gymnodinioid dinoflagellates). 304 amplicons were successfully direct-sequenced with sufficient quality to act as barcodes. Of these, 293 were included for barcoding analysis (the others being determined to be non-dinoflagellate sequences), together with 62 publicly available dinoflagellate COI sequences from Genbank. This resulted in a total of 336 sequences, representing 54 named species and five *Symbiodinium* clades. Most culture collections were heavily biased towards photosynthetic, planktonic and toxic genera such as *Alexandrium*, *Scrippsiella*, *Karlodinium*, and *Karenia*. Another well represented taxon was *Symbiodinium*, a diverse genus divided into the so-called clades A–H originally based on small (SSU) ribosomal subunit phylogeny and later incorporated results from other DNA markers that resulted in the subdivision of clades into subclades or types, as reviewed in [47]. Species in the genus *Gymnodinium*, consist of *Gymnodinium sensu strictu* but this genus is also an umbrella term to describe several distantly related species [39], [48], [49]. A full list of cultivated taxa is shown in Table S1.

**Figure 1:**
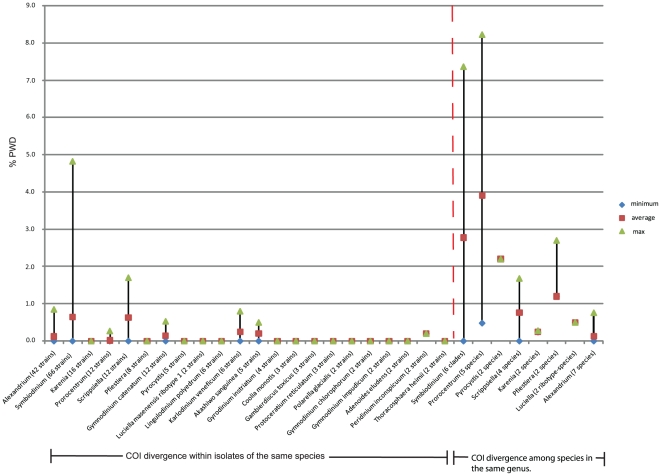
A Comparison of Uncorrected Pairwise Distances (PWD) of COI Barcodes from Selected Cultured Dinoflagellate Strains. Horizontal axis compares strain isolates within a species (or strains within a clade for *Symbiodinium*) with species (or *Symbiodinium* clades) within their genus, separated by a red dashed line. Most intra-species comparisons fall between 0 and 0.24%, except for two species, known to have high divergence or cryptic species. Note higher PWD values and variances obtained when comparing species within a genus, versus isolates within a species.

**Figure 2:**
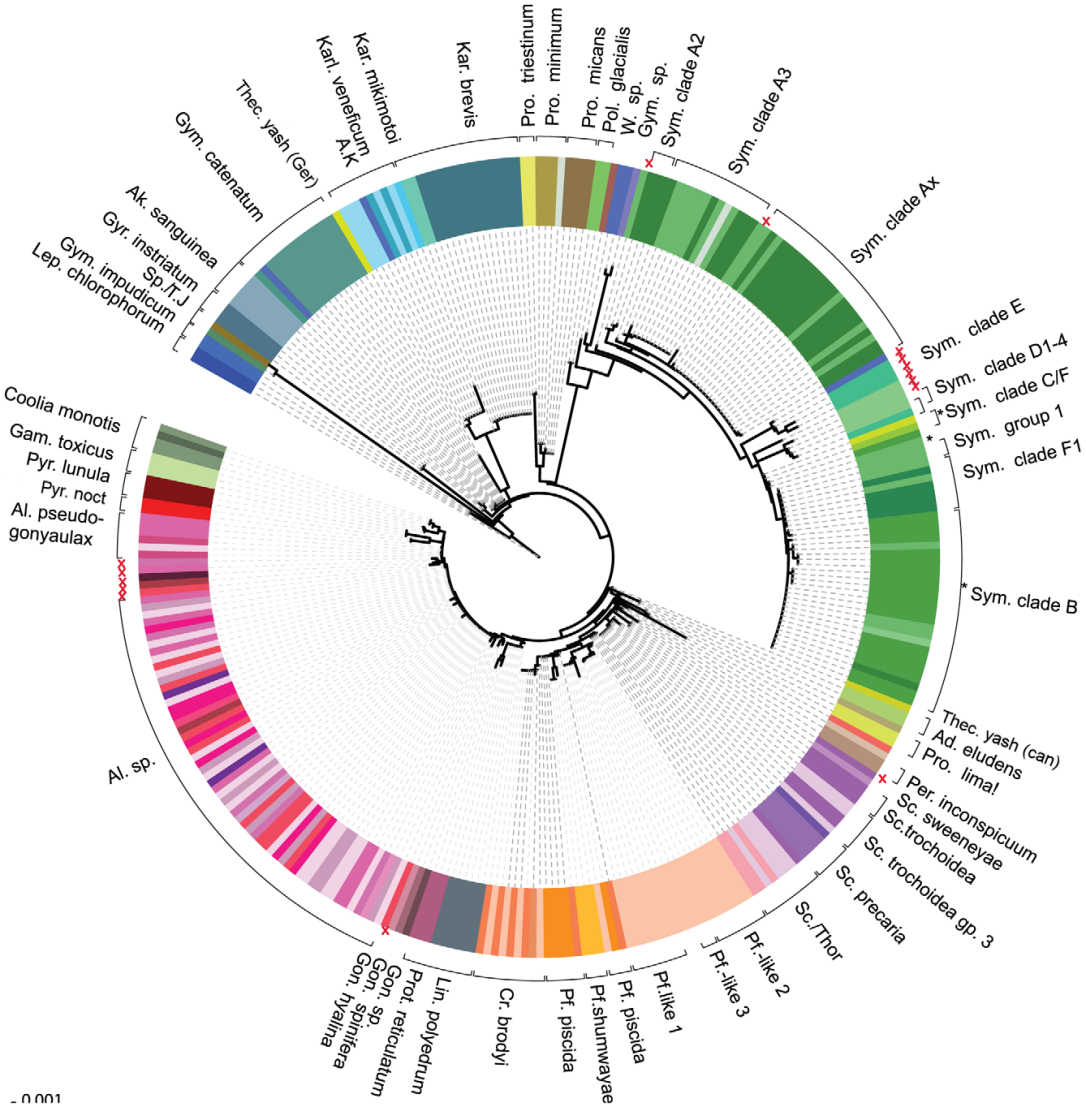
Neighbor Joining Cluster Analysis of Uncorrected PWD from all Culture Collection COI Barcodes. Each species is coloured according to its original species or clade designation. Species of the same genus or clades share a colour theme. Brackets indicate “barcode” species groupings, calculated by uncorrected pairwise distance of 0.24% or less, including those for *Symbiodinium* clades. Red crosses show strains that fall outside species range. Star symbols show strains that show species level similarity to more than one grouping, also marked similarly. Exclamation mark indicates the group is slightly above species cut-off threshold. Strain names were removed for clarity but are shown in Fig. S2. Species name abbreviations: A.K.: Antarctic Kareniaceae; Ad.: *Adenoides*; Al: *Alexandrium*; Ak: *Akashiwo*; Cr.: *Cryptoperidiniopsis*; Gam: *Gambierdiscus*; Gon: *Gonyaulax*; Gym: *Gymnodinium*; Gyr: *Gyrodinium*; Kar: *Karenia*; Karl: *Karlodinium*; Lep: *Lepidodinium*; Lin.: *Lingulodinium*; Per: *Peridinium*; Pf: *Pfiesteria*; Pol: *Polarella*; Pro: *Prorocentrum*; Prot.: *Protoceratium*; Pyr: *Pyrocystis* (*noct: noctiluca*); Sp: *Spiniferodinium*; Sc: *Scrippsiella*; Sym: *Symbiodinium*; T.J. *Togulla jolla*; Thec: *Thecadinium*. *T. yash* (Can) and *T. yash* (Ger) refer to Canadian and German isolates of *T. yashimaense* respectively. Thor: *Thoracosphaera*; W.: *Woloszynskia*. Unidentified cultured strains are shown in grey shading.

Average pairwise distances (PWD) were calculated for all strains with named species within 18 genera to measure variance within species over the whole dataset in order to account for differing sample sizes, ranging from 1 to 16 strains per species (average 3.4) and 6 *Symbiodinium* strains per clade. In calculating PWD that defined a species-O.T.U (Operational Taxonomic Unit), we excluded strains with no species names to retain objective comparisons with our COI-based findings, although this reduced our dataset. COI proved to be highly conserved and species names broadly agreed with COI barcodes across all culture collections. Nearly 73% of the strains could be assigned to a species at a value of 0.24% or less in eleven out of eighteen genera (see Fig. 1). At PWD of 0.5% or more, only 50% of strains actually corresponded to a particular species. However, when excluding *Alexandrium*, *Protoceratium*, *Lingulodinium* and *Symbiodinium* 81% of strains could be assigned to species because COI divergence rates in these genera were too low. We found that a PWD of 0.24% was a good cut-off value to determine species O.T.U groups by the neighbor-joining cluster analysis (Fig. 2 and Fig. S1), although this could exclude more divergent members of a species that occasionally arose such as *Symbiodinium* clade D strains (PWD = 4.6%) and *Scrippsiella trochoidea* (PWD = 1.7%). At these limits species and barcode definitions become challenging especially in cases of cryptic species, deep-lineage splits and morphologically similar but genetically divergent species.

Not surprisingly, the genus *Symbiodinium* showed the most sequence variation within species. As species designations are lacking for most specimens of *Symbiodinium*, these taxa are most commonly assembled into broad groups or clades that encompass many undescribed species (see [47], [50] for review and comprehensive phylogeny). Here we used our species O.T.U criteria from other dinoflagellate species to group *Symbiodinium* strains and found clade boundaries roughly equated with O.T.U criteria of non-*Symbiodinium* species, except for clade A (see below). Most *Alexandrium* species were identical in sequence to each other, with the exception of ten strains that are described in detail below. A lack of resolution of COI resulted in more than one named species belonging to one barcode O.T.U in strains for five genera.

Comparing species within a genus revealed large variation from zero to just over 8% (see Fig. 1). This probably reflects uneven sampling and relatively greater subjectivity in defining a genus compared to a species. The PWD range for inter-species comparisons was approximately bimodal with 65% of genera between 0–1.5% PWD range. Of this 84% of PWD values between 0–0.2% belonged to *Alexandrium*. Approximately 26% of PWD values occur between 3.3 and 5.9%, virtually all consisting of *Symbiodinium* (inter-clade comparisons) and *Prorocentrum.* For the latter, this genus was split between a benthic-living species group, *Prorocentrum lima* (0.27% similarity to *P. levis*), Prorocentrum levis, and a planktonic species group, *Prorocentrum minimum*, Prorocentrum micans and Prorocentrum *triestinum* with high divergence rates between these two groups. There is some debate regarding monophyly in this genus [51]–[53] that cannot be addressed using a DNA barcode. A similar pattern emerged between *Pyrocystis lunula* and *P. noctiluca* (PWD = 2.2%). *Prorocentrum, Pyrocystis* and *Symbiodinium* genera are outliers. Thus, PWD values from these genera were not used to estimate genus-level similarity for environmental barcodes (see below) because at this range, the definition of genus is tentative using DNA barcoding. Likewise, we also took account of bias from *Alexandrium* genus to skew genus-level identity to lower values and used 1.4% as an average of lower genus range to assess environmental barcode genera in order to avoid overestimation of species within a genus category and misidentification [54]. This value was in good agreement with macroalgal species-genera comparisons [22]. Ideally a barcode marker should be well represented in databases, be able to universally amplify all taxa and have enough sequence resolution to distinguish taxa to the species level. Overall, COI is too highly conserved to meet the latter criterion, but does nevertheless possess sufficient information to 70–75% of the accepted species represented in our survey of cultured strains.

#### Identification of misclassified and unclassified strains

Many strains in culture collections have not been identified to the level of species, and our analyses have successfully identified 101 of these to known species. Excluding strains that were refractory to COI barcoding due to poor resolution (see below), differences between barcodes and species or genus identity were encountered in 17 strains (see Table S1). The most significant discrepancies were found in *Symbiodinium*, *Alexandrium*, and Pfiesteriaceae.

Within the recently identified Pfiesteria group that inhabits brackish water [55] and includes *Pfiesteria piscida* Steidinger et Burkholder [55], *Pfiesteria shumwayae* Glasgow et Burkholder [56], *Cryptoperidiniopsis brodyi*, Steidinger et Litaker [57] and *Pfiesteria*-like group, many of which have been renamed as ribotypes of *Luciella masanensis*, *L. atlantis* [58] or *Stoeckeria* [59], we found seven strains that were mis-identified, (see Fig. 2 and Table S1). This was not surprising due to the difficulty in recognizing morphological features [60]. Four “*Pfiesteria*-like” strains were re-assigned to *Cryptoperidiniopsis brodyi*. Two *Cryptoperidiniopsis* sp. and a *Pfiesteria*-like strain were identified as *Pfiesteria piscida*. The *Cryptoperidiniopsis* strain CCMP1828 may contain more than one species as Steidinger and colleagues indicate there are discrepancies depending on where it is tested [57]. The 23 *Pfiesteria*-like named strains in our dataset (all from CCMP), these sub-clustered into three groups in a monophyletic manner. Out of these, six more strains of *L. masanensis* ribotype type 1 and three strains of ribotype type 3 were identified, including two originally described by Mason and colleagues [58] and all from Priest landing Wilmington River, GA. Ribotype 3 has not previously been reported from that location by these authors. Two strains belonged to a third, unknown *Pfiesteria*-like group whilst three strains, CCMP1845, CCMP1880 and CCMP2840 (all from different locations), showed similarity to more than one Pfiesteriaceae group. Strain CCMP2840 (GQ501207) from Cedar Island (NC, USA) showed species-O.T.U identity to both *Pfiesteria* and *Thoracosphaera* genera. These results may indicate lack of resolution, but one strain, CCMP2182 (GQ501273), isolated from ballast water, could not be assigned to *Pfiesteria* or *Cryptoperidiniopsis* as it was heterozygous in three positions that differentiated the two species and may comprise a mixture of *Pfiesteria* and a *Cryptoperidiniopsis*. None of these undetermined *Pfiesteria*-like strains could be confirmed as *L. masanensis*, *L. atlantis* or *Stoeckeria* as these strains have not been described elsewhere to our knowledge.

Cryptic diversity is also evident within *Scrippsiella*, with a core *S. trochoidea* group distinct at species O.T.U level from a second *S. trochoidea* strain group (called group 3). One *S. trochoidea* strain (GQ501326) may be misidentified as it grouped within a non-specific *Scrippsiella/Thoracosphaera* group, rather than any *S. trochoidea* groups. CCMP2775 (GQ501330) had greater than genera-level O.T.U distance to any other *Scrippsiella* or *Thoracosphaera* species and so was labeled Calciodinellaceae. The separation of *S. trochoidea* strains is consistent with the description of distinct ‘*S.trochoidea*’ species complexes using ITS sequencing [61], [62]. Four *S. precaria* strains and one *S. cf lachrymosa* strain were identical to each other and had a conserved nucleotide not present in other *Scrippsiella* sp. However, *Thoracosphaera heimii* and *Scrippsiella* sp. are represented in one species O.T.U clade in our analysis. These strains cannot be resolved in species groups using COI barcodes.

In other cases, taxonomically distinct species were found to have identical DNA barcodes. In our dataset, eight species showed no distinction by COI barcode. *Karenia mikimotoi* and *K. brevis*; *L. polyedrum* and *P. reticulatum*; some of the aforementioned *Scrippsiella* and *Thoracosphaera* plus *Togulla jolla* (HM236201) and *Spiniferodinium* (GQ501332). The latter two are both of uncertain taxonomic position but separated at the genus level and unlikely to be the same, as they possess different morphologies. In addition, nine different *Alexandrium* species had identical barcodes. Ten strains were found to have unusually high levels of divergence compared to the rest of the genus. Of these, six could be identified as *A. pseudogonyaulax*, and the remaining four could not be placed within any known clade. One identified *A. pseudogonlaulax* strain, CCAP 1119/1, may be a contamination as it is named *A. tamarense* and three of its strain synonyms were different to it. The latter strains may need taxonomic revision given their unusually high species-O.T.U divergence levels compared with those of other *Alexandrium* species that were identical by COI barcode sequence.

In contrast, other taxonomically related species were found to be quite distinct. The two *Thecadinium* species analysed here did not cluster together: *Thecadinium yashimaense* (as *T. inclinatum*) (HM236199) collected from Canada (held in CCCM) was later identified as *T. mucosum* [63] and was subsequently re-classified as conspecific to *T. yashimaense* and *T. fovealatum* [64]. However, our data showed that *T. yashimaense* (inclinatum) (HM236199) and *T. yashimaense* (HM236200) from Germany were distinct species. As there are no molecular data for *T. yashimaense*, these two strains may be two different species of *Thecadinium* that may have originally been misclassified. Small cells interpreted as life cycle stages were observed in the cultures (unpubl. data), so there is the possibility of a mixed culture of the two species.

#### *Symbiodinium* Identification

**Figure 3:**
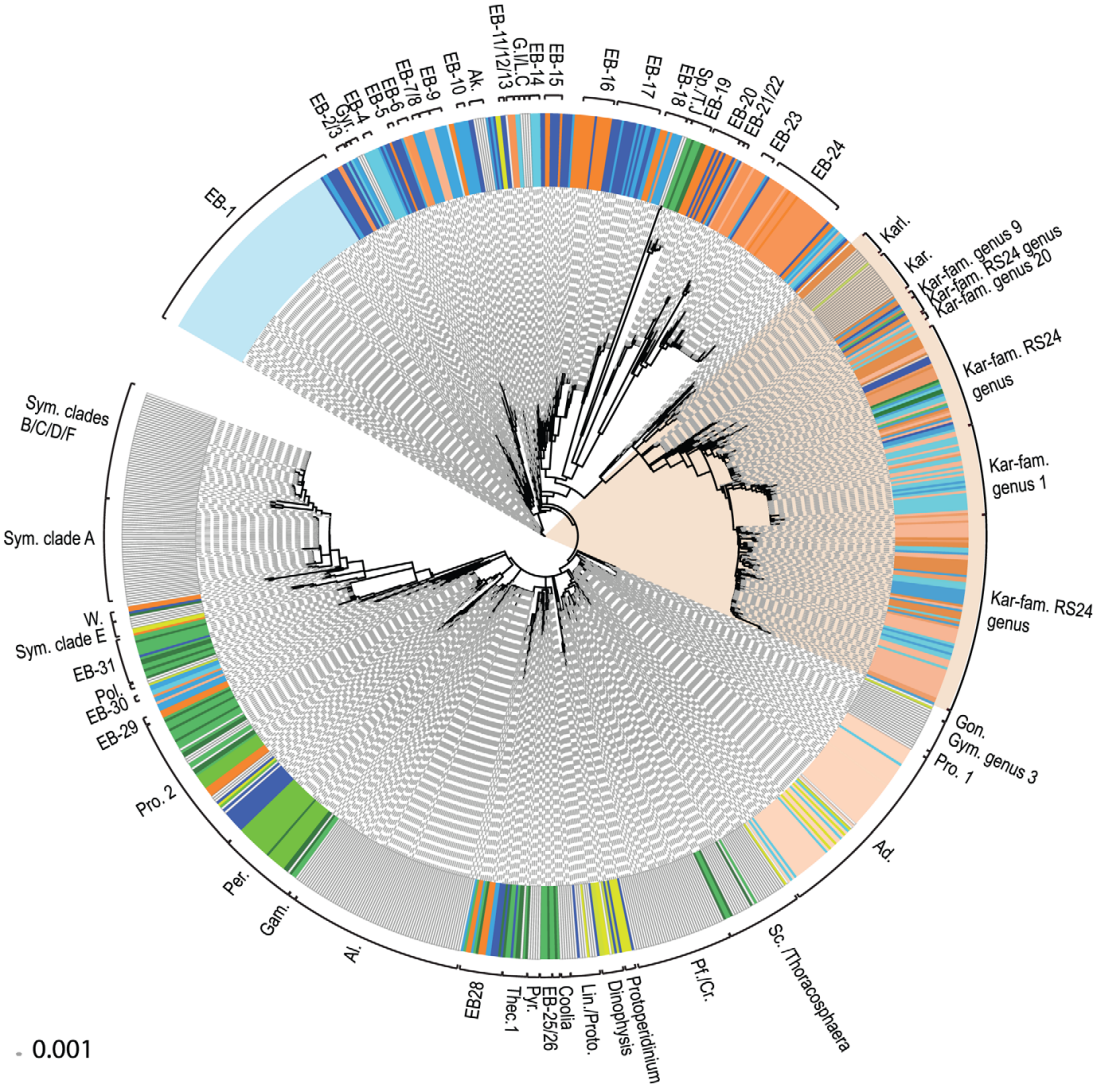
Compiled Neighbor Joining Cluster Analysis of Uncorrected PWD from Both Culture Collection and Marine Environmental COI Barcodes Reveal High Levels of Diversity. Brackets indicate all strains within a minimal barcode genus-O.T.U, with a PWD cut off of 1.4%. Pink shaded area indicates strains belonging to the Kareniaceae family. Where environmental barcodes (EB), could not be identified to any known strain in our database, they were labeled with a numbered EB genus, except for strains belonging to Kareniaceae family where they were prefixed Kar-fam., and then an assigned genus-O.T.U number or the name of most related known strain. All strains are listed in Table S1. Clear bars represent known taxa included in Fig.2, G.I/L.C: *Gymnodinium impudicum* and *Lepidodinium chlorophorum*. Coloured strains represent EBs, where red indicates Saanich Inlet in summer and blue represent Winter Saanich Inlet EBs. The colour shading in each group is proportional to sampling depths, from lightest to darkest indicating 10 m, 100 m, 150 m, 200 m respectively. Green shaded strains come from the Maine coast, red shaded strains are from the Caribbean and yellow shaded strains are single cell barcodes. Red arc shading shows the extent of species expansion from Kareniaceae family. Strain names were removed for clarity but are shown in Figure S2.

Takabayashi and colleagues [65] found that COI markers corresponded well with *Symbiodinium* clades using a nuclear marker and chloroplast marker (Cp23S-rDNA) [66]. A total of 81 *Symbiodinium* strains could be classified to their correct clade or subclade using shorter COI barcodes and an additional 23 previously strains that were unclassified or had ambiguous clade status were successfully attributed to a clade. Of these, 64 strains had no species/clade identity, two were completely unidentified and three were misclassified. Furthermore, our genus-level O.T.U based on these barcodes are congruent with the authors' principle groupings (Fig. 3). *Symbiodinium* DNA barcodes formed eight clusters (Fig. 2) that corresponded well with clades B, D, and F and within genera-level O.T.U for clade E (0.3–1.4%). Clade A strains were reliably identified at the clade level, but the subgroups of clade A only partially overlapped with subclades as defined by LaJeunesse [59].

Group A3 included two sequences belonging to clade A3, but also contained two strains that were identified to Cp23S-rDNA genotype marker A194 (nomenclature based on clade A and size of the hypervariable region, 194 bp) that generally corresponds to A1 but, may also belong to clade A4. Group Ax contained a mixture of strains belonging to A194 and a second, uncharacterized Cp23S-rDNA clade A genotype called A188 and contained all full-length COI clade A sequences deposited in Genbank. Strain Zs (AY289692) was identical to CCMP2461 (GQ501337) and had borderline identity (0.24–0.27%) to CCMP2429 (GQ501395), a more distal clade A2 strain ([67] and R. Moore, personal communication).

Clade C/F contained two strains, CCMP2466 (GQ501334 clade C1) and Mv (AY289712) originally identified as F1 [68] but now designated to clade F5 [50]. This group is given provisional C/F status and probably grouped together due to lack of COI resolution. Both these strains were identical to CCMP2434 (GQ501353) and have borderline clade identity (0.24%) to members of clade B and F, although CCMP2434 (GQ501353) is identical to just under half of clade B strains. The strains in clade C/F are clearly distinct but related to either clade F or B so CCMP2434 (GQ501353) may be a B subtype. Although most members of clade F and B were easy to distinguish, a previous study showed AY289712 (strain Mv) belonged to clade F but shared an identical Cp23S-rDNA genotype allele size with clade B [66]. Subsequent sequence analysis revealed these to be two distinct strains, but such homoplasy may be replicated using COI barcodes in these strains. MAC-Pdiv 45a (GQ501370) had ambiguous clade identity (either F or B), but was identified as clade B using COI.

*Symbiodinium* clade E [68]–[70] gave unexpected results, especially for the three strain synonyms of CCMP421 (GQ501340, GQ501339, GQ501242), belonging to clade E2, and an unclassified strain attributed to *Gymnodinium* (AC561, GQ501241) which were between 0.3–0.9% similar and well within genus level O.T.U of each other. The identification of AC561 is significant as there are only two other clade E cultures. In particular, nucleotide variation was observed in six positions over a 60 bp region in all CCMP421 strain synonyms barring AC561. CCMP421 was started as a single cell and was previously misidentified as *Gymnodinium* [69]. A fourth strain synonymous to CCMP421 deposited in Genbank (AY289708) did not fall within clade E with the other synonyms, but instead showed borderline clade level identity to one of the two clade D1-4 (“*S. trenchi*”) strains (GQ501397) [71]–[73] which also clustered together with full length COI gene [65]. The placement of CCMP421 strain AY289708 in clade D may be because this sequence is a COI paralogue (see below) or because of phylogenetic uncertainty between clades E and D using COI in some studies ([65] and reviewed by [74]). Two previously identified clade D strains, PSP1-05 and HpiH-showed no identity to any strain in our database. PSP1-05, is recognized as a highly divergent member of clade D1 [65], [69] so this result is not surprising.

### Testing for paralogues and pseudogenes

Pseudogenes are known to occur in dinoflagellates [66], [75], their organellar genomes are highly unusual and their mitochondria contain many fragmented gene copies which could be co-amplified in this instance [76], [77]. To determine whether this was the cause of unusually high variation observed in strains belonging to *Scrippsiella trochoidea*, *Prorocentrum* and *Symbiodinium*, we sequenced 30 clones from seven strains (average 5 clones per strain) belonging to *Scrippsiella* sp. (2 strains), *Prorocentrum* (3 strains) and *Symbiodinium* sp. (2 strains, including CCMP421). Comparing the directly sequenced strains with their cloned counterparts (see Table S3) showed very slight variation, none more than 0.2% PWD for all but one strain, less than the species-O.T.U cut off of 0.24% indicating strain diversity has not been overestimated in these species. No clones of cryptic *S. trochoidea* strain CCAP1134/9 overlapped with separate *S. trochoidea* (CS-297). The one exception to this were clonal DNA barcodes of CCMP421, where the average distance between all clones plus the direct sequence was 1.3%, a value expected for an average intra-species comparison and likely to be due to the presence of multiple paralogues. Three CCMP421 COI barcode clones were identical to that of AC561, which explains at least some of the variation observed in the directly sequenced strain synonyms of CCMP421. No stop codons were present in cloned and directly sequenced barcodes except for CCAP 1136/16, *Prorocentrum minimum* (GQ501297) at positions corresponding to previously reported RNA editing sites for this species [78].

### Comparing culture collections to natural diversity

In order to evaluate how much natural diversity is represented in culture collections, environmental DNA barcoding of total planktonic DNA from three different marine environments was carried out with dinoflagellate-specific primer sets. The deepest sampling was done from Saanich Inlet - a marine fjord off Vancouver Island that is hypoxic from 100 m to 200 m depth in the summer, but which is mixed in the winter. Near-coastal planktonic samples were also taken from the coast of Maine, USA and the island of Guadeloupe in the Caribbean. After screening out poor quality sequences, this resulted in a total of 713 environmental barcode sequences (listed in Table S2): 574 barcodes from Saanich Inlet (Northeast Pacific); 86 from Maine (Northwest Atlantic), and 29 from Guadeloupe (Caribbean). Finally, to show that both morphology and DNA barcodes could be recorded for single cells, especially those refractory to culturing, we also isolated 24 single dinoflagellate cells from the west coast of Vancouver Island, photographed them, and produced COI barcodes from individual cells. Photographed single cells were also used to corroborate the identity of unknown environmental barcodes belonging to the same species-O.T.U.

Clustering the combined culture collection and environmental data resulted in a total of 1049 dinoflagellate barcodes (Fig. 3) that showed depth and seasonal stratification (Fig. 3) also confirmed using Principle Coordinates Analysis (PCoA) (Fig. S3). The most striking first observation from these data was the level of diversity within the environmental groups with 531 different barcode species out of 603 unique environmental barcodes, mostly from Saanich Inlet. Although this study only covered 54% of known cultured strains, it is still striking that only 91 of those environmental barcodes, including single cells could be matched at the species level (261 at genus level) to cultured strains using an average cut off value of 0.24%. This Figure fell to 51 identified barcode clones, once identical clones were removed. A further 92 unique environmental barcodes that were related to each other could not be correlated to any cultured species. In total, 24% of unique environmental barcodes were related to at least one other sampled sequence from a known or unknown strain at the species level. To link environmental barcodes to known phylotypes at a broader level, each environmental barcode was binned with a known genus if it was within the PWD boundaries defining that genus (this varied between genera as each had different levels of sequence diversity) plus to any other strains also grouped with that genus. By these criteria, many environmental barcodes could not be identified to any known taxon at any level, so in these cases an average genus-level PWD cut-off of 1.4% was used. This was a conservative estimate, in line with minimum values for most of the known genera. Based on this genus-level PWD estimate, environmental barcodes were grouped in the cluster analysis shown in Fig. 3, except for the highlighted grouping containing *Karlodinium, Karenia* and an unnamed Antarctic dinoflagellate (RS24) belonging to the Kareniaceae family [79], [80] which is an exceptional case discussed below. In addition to the Kareniaceae, the second major identified group in the environmental samples are a group of sequences identical or closely related to *Adenoides* or *Amphidinium cf. semilunatum* [81]. These occurred exclusively in 10 m July 2006 Saanich Inlet samples and matched cultured in single cell isolates, whereas an unknown cluster (shown in pale blue, Fig. 3, and blue circle in Fig. S3) represented almost the entire diversity of 10 m winter sample from Saanich Inlet. These are the only seasonally related clusters. Many of the Caribbean and Maine samples grouped with *Prorocentrum* and *Peridinium* sp. and a minority to *Scrippsiella* or *Thoracosphaera*. Finally a small number of environmental barcodes, mostly from unidentified groups EB23 and EB24 (Fig. 3 and Fig. S2) were found exclusively in deep water, from 100–120 m depth in Saanich Inlet exclusively in summer, below the hypoxic boundary.

Overall, the majority of environmental barcodes from these marine samples could not be identified because there was little overlap in diversity between the environmental and cultured dinoflagellate barcodes. In addition, there was little overlap of species assemblages between different environments. Nevertheless numerous other environmental barcodes could be identified to the genus level or higher, and many of these represent great expansions in the known diversity of these groups.

#### Single-cell barcoding

**Figure 4:**
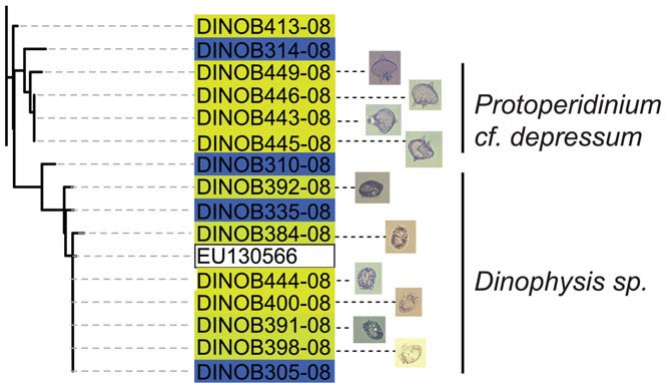
Expanded View of Neighbor Joining Cluster Analysis from **Figure 3**. Showing *Dinophysis* and *Protoperidinium* Single Cell Barcodes with Their Photomicrographs. Black solid lines show species level similarity.

With the majority of environmental barcodes not attributable to any taxonomic group represented in the barcode library of culture collections, a method to connect environmental barcodes to cells will be required to assess natural microbial diversity. For a start, barcoding from single cells that have been photographed prior to isolation would at least allow us to identify major groups otherwise only made up of environmental sequences. To test this in principle, almost 70 single cells were photographed and manually isolated from the west coast of Vancouver Island, and 24 COI barcodes were generated from these single cells, although images for four single-cells in this study (GQ502036, HM236194, GQ502039, HM236195, GQ501405) were very poor quality and discarded. The single-cell barcodes proved to be successful in providing benchmarks for the environmental barcode libraries for the difficult-to-culture heterotrophic *Dinophysis* and *Protoperidinium* genera (Fig. 4), as well as identifying representatives of other major environmental groups with poor representation in culture collections such as *Adenoides* (GQ501403-GQ501406) which is found in benthic and seawater environments. These strains, together with cultured *Adenoides eludens* strains were used to aid the identification of a large cluster of uncultured environmental barcodes from Saanich Inlet (2006), almost exclusively found at 10 m. GQ501404 was morphologically similar to the taxonomically unresolved *Amphidinium semilunatum*, a species that is distinct from *Adenoides*, but both previously grouped under the genus *Amphidinium*. GQ501404 was identical to cultured *A. eludens* so it is possible that the COI barcode lacks resolution to distinguish between these two potentially separate species. However, GQ501404 had eight non-ambiguous sequence differences to the COI barcode of a third *Adenoides* species, NIES-1402 (HM355857-not included here), that suggests this group requires taxonomic re-evaluation.

#### Kareniaceae – a case study of exceptional natural diversity

Only four species of Kareniaceae are represented in our barcodes from culture collections (Fig. 2), but the diversity of this group exploded with the addition of environmental barcodes from the Northwest Atlantic, and particularly from the Northeast Pacific.

The Kareniaceae have traditionally been a relatively small family made up of dinoflagellates that are distinguished by having a 19′ hexanoyloxyfucoxanthin-type plastid derived from a haptophyte [82]. The Antarctic dinoflagellate RS24 (GQ501253) is a sister species to *Karenia* and *Karlodinium* [80], with a temporary haptophyte resident identical to *Phaeocystis antarctica*. [79]. Recent studies have shown that Kareniaceae are more diverse than previously thought, with another six new species recently described [83] but there still only 20 known species in this family, including the RS24. However, of 713 environmental sequences, 177 clones fell within the Kareniaceae group as a whole, and 122 clones representing 88 species showed genus-O.T.U level identity with RS24 that showed same grouping with PCoA (Fig. 3, Fig. S3 and Table S2). These included 26 environmental barcode clones mostly from Saanich Inlet which had species-level identity to RS24 (Fig. 3) and this species was the most common among the Kareniaceae. One non-photosynthetic single-cell PL9-11 (GQ502034), was found to belong to this family but was genetically different to RS24, and may also represent another species Interestingly, virtually all environmental barcodes falling within this group were from Saanich Inlet and from depths between 100–120 m, although there were no distinct seasonal or habitat differences, with barcodes clustering with RS24 at species level being found in Northwest Atlantic too.

## Discussion

### Barcoding culture collection strains

Culture collections represent the most accessible and traceable repositories of living microalgae, but they are also biased towards the species and strains most amenable to cultivation, and/or of commercial and medical importance. It has long been known, especially in prokaryotes, that only a small fraction of natural diversity is easily cultured using common strategies. Accordingly, cultured strains do not adequately represent either the depth or breadth of microbial diversity, but they still make useful benchmarks for molecular barcode databases of natural diversity. However, while it is becoming increasingly easy to use molecular data for taxonomic surveys that avoid culture work, cultures still remain a primary source for scientists who need cells for biochemical, biotechnical, cellular and physiological studies. These scientists will benefit greatly from barcodes that can provide an easy and quick means for quality control.

COI barcoding worked as a means of distinguishing most dinoflagellate species in 15 of 21 genera where COI sequence divergence rates were congruent with speciation events. For those 15 genera 81% of strains could be distinguished at species-O.T.U level using a PWD cut-off value of 0.24%. This criterion would allow for the majority of COI samples obtained from environmental samples to be binned into genetic clusters which on average corresponds with distinct species. This approach will overestimate the number of true species in genera with higher divergence rates and underestimate those with lower rates of divergence, but on average it should give a reasonable estimate of species diversity. A small assessment of species in the most variable genera demonstrated that genetic differences are unlikely to be due to pseudogenes or paralogues in all but one strain (discussed below), at least for those species tested. Other species, such as those belonging to Kareniaceae, showed low intra-species PWDs and unlikely to possess paralogues that would artificially inflate observed diversity in the environmental barcode dataset.

Assessing species diversity in the five genera (*Alexandrium*, *Lingulodinium*, *Protoceratium,* some *Thoracosphaera* and *Scrippsiella* sp.) where COI sequence divergences were too highly conserved or too divergent (*Symbiodinium*) to prove useful in distinguishing species will require a different barcoding marker. For example, our failure to find a robust breakdown of *Alexandrium* strains in common with *cob* barcoding study by Lin and colleagues [35] reflects a well-documented problem with this genus, whose morphology can vary greatly under different environmental conditions [28], [84]–[87]. Due to this difficulty, several species have been grouped into species complexes (reviewed in [28]). However, the correct choice of marker may allow resolution of taxonomical challenges, such as the successful use of the variable region of the large ribosomal subunit to identify cryptic species within the *A. tamarense* species complex [88]. Despite this lack of complete coverage, the COI can be used to assess species diversity in a number of important dinoflagellate groups such as *Karenia* and *Karlodinium* genera. Systematic barcoding of available cultures has revealed instances of out-dated nomenclature, cryptic diversity, and misidentifications, all of which may be expected in such a diverse and abundant group. The advantages of DNA barcoding allows for quick, cost effective means of identifying species that can be carried out using easy-to-use and fast phylogenetic and bioinformatic tools, which will become easier as the database expands. The COI marker has the advantages of circumventing time-consuming and expensive cloning procedures.

It is difficult to compare the effectiveness of COI in comparison to cob [35], with two different methods of analysis, different sampling depths and different species studied. However, comparing similar species groups, both COI and cob showed little intra-specific variation, and inter-specific variation of COI in our studies were remarkably similar to that of cob, except for *Symbiodinium* which had a much larger range in our study, due to the larger sample size of our dataset. The *cob* gene was found to resolve about half of the diversity in a study by Sampayo and colleagues and was not suitable for ancestral *Symbiodinium* types [89]. As a whole, these results were unexpected given that cob was reported to be more variable than COI but could be explained, in part, by the greater sampling depth of known species used here.

#### DNA barcoding of a diverse genus: *Symbiodinium*

The genus *Symbiodinium* has largely been recognized to be incongruent with other dinoflagellate genera in terms of genetic diversity. COI barcoding made largely accurate identifications of the major clades in accordance with *Symbiodinium* phylogenies [47], [50], [65]. However, the lack of fine level resolution with COI, and the short length of a single DNA barcode is unlikely to capture accurate phylogenetic relationships achievable with longer markers [69], [70]. Therefore the subclade groupings did not correspond entirely to those described by LaJeunesse [68], particularly for subclade A, and is probably the reason for the ambiguous identity of some strains in clade F, C and B. COI barcodes would be useful as a complementary marker for example, resolving the identification of two strains with ambiguous clade identity. The variation observed in multiple CCMP421 strain synonyms appears to be caused by the presence of multiple paralogues, which was not observed in MAC-579 (clade B). An unusually high number of ITS paralogues have also been reported for CCMP421 [90] which may have an evolutionary significance that deserves further investigation.

Whilst monoclonal cultured cells can be a useful source to control morphological plasticity [68], one caveat of using cultured *Symbiodinium* for identification is they may not reflect the true biological symbiotic strain in the host. Often, cultures can contain surface contaminants such as free-living forms of *Symbiodinium* [68], [91]. COI barcodes have uncovered strain misidentifications and also a rare new clade E culture (AC561) and highlight the usefulness of DNA barcoding in culture collections. Although COI is not suitable to identify *Symbiodinium* strains to subclade and type level, it could be considered an easily amplifiable validation marker for *Symbiodinium* diversity in the same way as other organelle markers have, namely *cob* and Cp-23S rDNA (chloroplast large subunit rDNA gene). The latter were able to identify strains to species level when combined with nuclear markers [89]. This could reduce the considerable problems caused by ITS paralogues and pseudogenes [90]. Furthermore, these classifications could allow more universal comparisons with other dinoflagellates in future.

### Diversity of dinoflagellates in marine environments

In common with previous SSU deep amplicon sequencing surveys of protists in marine environments [17], [18], our study revealed astonishing diversity, although SSU may be too conserved to distinguish many species of dinoflagellates [92]. However, diversity may not exclude ubiquity as environmental diversity studies have identified the same species in distant regions [18], [35]. The Saanich Inlet fjord system has many discrete habitats: the summer thermocline in Saanich Inlet and an additional halocline from glacial waters mixing at the surface creates ecological boundaries, partitioning species into ecological niches that may support genetically stratified populations of dinoflagellates by sexual and/or environmental selection. This dataset revealed massive diversity in environmental dinoflagellate barcodes at 10 m and 100 m in summer and winter, which showed spatial and temporal species-level separation. Do our results indicate cosmopolitan or endemic species? The diversity in Kareniaceae family, the lack of overlap of some environmental sequences at different depths and seasons give some indication of endemism. Although very little overlap existed between the three environments, there was evidence for cosmopolitan species: the Antarctic *Karlodinium*-like dinoflagellate, RS24, and some *Scrippsiella* sp. were common to NE pacific and NW Atlantic samples. Relatively few sequences were obtained from of NW Atlantic and Caribbean environments so with greater sampling depth there could potentially be more species that overlap between different environments. The nature of the environment is a major force that selects for endemic or cosmopolitan species. Deep sequencing of ecologically similar environments in different locations using DNA barcoding methodology will be instrumental in addressing this debate. Without morphology to aid identification, studies should consider confounding factors such as paralogues, by using more than one marker. A more difficult question is how to apply cut off values in order to accurately measure species diversity, given uneven speciation rates.

#### Kareniaceae diversity

Recent studies described five new species of Kareniaceae [83], in addition to RS24 described by Gast *et al.* [79], although all at surface waters. There are many toxic species in this family, yet little is known of them because they are small, fragile and poorly sampled. A large proportion of these dinoflagellates existed at 100 m and lower. It is unclear how these and the two other groups of deep water dinoflagellates survive at these depths, some below the hypoxic boundary. Zaikova and colleagues [93], [94] analysed the same Saanich Inlet samples for bacterial diversity and reported a dominant bacteria group, SUP05 present at 100 m and below in 2006 for both seasons. SUP05 was originally identified from Suiyo Seamount [95], and a common hypoxic water species which may be a food source for these dinoflagellates. Deep water heterotrophic Gymnodinioid species have also been identified in deep, stratified marine systems such as the Gulf of Gdansk (over 100 m) [95]. Alternatively they could be cysts, although cysts have not been described in *Karlodinium* or *Karenia* [96]. Our findings have contributed to evidence of a diverse and uncharacterized group of deep water dinoflagellates from stratified marine waters that have adapted to this habitat, possibly with unique trophic and respiratory mechanisms.

#### Seasonal diversity in Saanich Inlet

The summer and winter species compositions in Saanich Inlet were distinct and dominated by one genus. Coincidentally the greatest different in abiotic measurements were found between the February and July time points [93]. Forty four percent of the summer 10 m samples contained photosynthetic *Adenoides*, a genus with only two known species ([81] and unpublished data) that increased to 35 species-O.T.Us using our criteria. The February 2006 dinoflagellates at 10 m were unidentifiable but again were diverse,numbering 61 species-O.T.Us. A study of winter phytoplankton from 0–20 m depth in 1978 by Takahashi and colleagues [97] revealed dinoflagellates were the second dominant group, mostly consisting of *Katodinium rotundum* where light and temperature were the predominant limiting factors for growth. Given the dominance of Gymnodinioid dinoflagellates in both our samples and that of Takahashi *et al.* [97] perhaps *K. rotundum* may belong to the 10 m Saanich Inlet winter surface dinoflagellate assemblage and deserves further investigation. The use of single-cell barcoding would be particularly useful in identifying this and other unculturable protists and could be automated by flow cytometry, which has already been applied to protists [98] and provide better estimates of natural diversity in these communities.

### Concluding Remarks: natural diversity of microbial life

Our results show cultured dinoflagellates that are considered to be different species can be resolved using DNA barcoding, although robust taxonomy using other DNA markers, morphology and chemotaxonomic markers such as lipids and pigments is needed to provide a solid basis for DNA barcoding to work [99]. The vast majority of cultured species studied here could be identified with a PWD of 0.24% or less, showing that the concept of DNA barcoding can work for dinoflagellates and could be used to identify and segregate taxonomic units in environmental studies, although COI might not be the best single gene with which to assess and identify dinoflagellates. The true value of barcoding is its scale and ease to match unknown environmental sequences with a single cell barcode or a culture collection strain.

The environmental barcodes in this dataset may be a small fraction of the real diversity of the environments they represent, since none seemed to be sampled exhaustively. An even tinier proportion of dinoflagellates are represented by the combined holdings of culture collections, reflecting human bias in sampling and cultivation. DNA barcoding studies will be instrumental in evaluating biogeographical speciation and ecological assemblages within protist populations. With increasing use of next generation sequencing technology that can combine multiple markers, deep-level biodiversity studies will be more able to demonstrate true estimates of protist diversity and may provide useful information for the cultivation of a greater proportion of presently unculturable species. These methods combined with flow cytometry, already in use [[98], [100], [101], would provide additional morphological characters enabling single cells from environment and cultures to be evaluated at the individual rather than population level.

## Materials and Methods

### Sample Collection

Cultured strains or DNA samples were donated or purchased from eight public and three private culture collections, summarized in Table S1. Summary codes for strain donors are thus: AC: Algobank-Caen, Université de Caen Basse-Normandie, France; CCAP: Culture Collection of Algae and Protozoa, Scottish Association for Marine Science, U.K.; CCMP: Provasoli-Guillard National Center for Culture of Marine Phytoplankton, Bigelow Laboratory, USA; CS: Australian National Algae Culture Collection, CSIRO, Australia; CAWD: Cawthron Institute, Culture Collection of Micro-algae (CICCM), Nelson, NZ; NEPCC: North-East Pacific Culture Collection (part of Canadian Centre for Cultured Microorganisms, CCCM), University of British Columbia, Canada; NIES: National Institute for Environmental Studies, Japan; BURR: Buffalo Undersea Reef Research Culture Collection, State University of New York at Buffalo, Buffalo, USA MH: Mona Hoppenrath, Forschungsinstitut Senckenberg, Germany; UTEX: The Culture Collection of Algae at the University of Texas, Austin, TX, USA. Culture RS24 was donated by Rebecca Gast from Antarctic Protist Culture Collection, Woods Hole Oceanographic Institution, Woods Hole, MA, USA (private).

For environmental analysis, planktonic samples were collected through a 20 m plankton net taken from the island of Guadeloupe in the Caribbean (15.1539N, 61.3475W), from the Bigelow Laboratory pier, West Boothbay Harbor, ME, USA (38.1904N, 76.2707W) and also from mouth of the Damarascotta River, Maine, USA approximately 44N, 69.5W) representing two different coastal environments in Maine, USA (Northwest Atlantic). DNA from Saanich Inlet, Vancouver Island, BC, Canada (43.39N, 123.39W) (Northeast Pacific) was kindly donated by Dr. D. Walsh and Dr. S. Hallam, UBC, from non-filtered marine water collected by David Walsh in water collector containers at depths of 10 m, 100 m, 120 m and the bottom at 200 m in equal volumes. These same samples are also described by [93]. Single cells were collected from surface plankton or sand in Bamfield, Vancouver Island, BC, Canada; Saanich Inlet, BC, Canada. Isolation was performed under an inverted microscope. Cells were picked using a sterile extended pasteur pipette from a dish, washed in fresh autoclaved seawater to check for single isolation under a microscope, photographed and picked using a separate autoclaved, pipette. Benthic samples were filtered from sand using a method described by Hoppenrath and Leander [102] and single cell isolated from these samples were processed in a similar manner to those of planktonic samples. All uncultured, environmental samples used in this study are listed in Table S2.

### DNA extraction

Typically between 1.5–15 ml of dinoflagellate cells from culture were collected by centrifugation at initially 3000 g then at 1150 g, snap frozen in liquid nitrogen and thawed three times. For one third of culture collection samples, additional grinding was performed using plastic pestle and microfuge tube. DNA extraction was carried out using the DNeasy™ plant purification DNA kit (Qiagen, Mississauga, ON, Canada), following their protocol except incubating cells in lysis solution for 30 minutes instead of 10 minutes. Masterpure™ Complete DNA and RNA Purification Kit (Epicentre® Biotechnologies, Madison, WI, USA) was also used in about one third of cultures and for single cells, using Lysis of Fluid sample protocol followed by Precipitation of Total DNA protocol. For whole marine extracts DNA extraction was performed by mixing whole marine sample with an equal volume of a phenol-chloroform- isoamyl alcohol mixture (25∶24∶1) (Sigma-Aldrich, Oakville, ON, Canada), the DNA containing phase was removed and DNA extracted with the addition of 2.5 volumes of 100% ethanol (Sigma- Aldrich, Oakville, ON, Canada) and 0.1 volumes of 3M sodium acetate, pH 5.2 (Sigma-Aldrich), washed twice in 75% Ethanol and resuspended to 300 µg/µl in sterile water.

### PCR, cloning and Sequencing

Highest amplification rates were achieved using a nested primer set, consisting of primers DINOCOX1F 5′AAAAATTGTAATCATAAACGCTTAGG 3′and DINOCOX1R TGTTGAGCCACCTATAGTAAACATTA described by [52] and then using a nested primer set designed by B. Imanian COX1.DINO.F 5′ GAATTTGGAGGTGGCACNGGNTGGACNYT 3′ and COX1.DINO.2.R 5′-CCCATCGTATACATRTGRTGNCCCCANAC 3′. PCR amplification was carried out on 25–100 ng of DNA using PuReTaq Ready-to-Go beads (GE Lifesciences, NJ, USA) at 94°C for 3 minutes followed by 35 cycles of 94°C for 30 seconds, 48°C for 30 seconds and 72°C for 45 seconds, ending with a 72°C extension step for 7 minutes. All culture collection and single-cell cultures were sequenced directly. Single PCR products were diluted to 30 ng/µl or purified by gel extraction using the QIAquick Gel Extraction kit (Qiagen, Mississauga, ON, Canada), according to manufacturer's instructions and either sent to Canadian Centre of DNA Barcoding, Guelph, ON for DNA sequencing or sequenced directly using BigDye v3.1 reagents on at NAPS unit at University of British Columbia, BC. COI amplification products from whole marine extracts, or environmental barcodes, were cloned using TOPO TA cloning kit (Invitrogen, Burlington, ON, Canada) according to manufacturer's directions except 200 µl of transformations were plated onto LB ampicillin plates. Transformed white colonies were screened using Amplitaq kit® (Invitrogen, Burlington, ON, Canada) as per manufacturer's instructions using 1 µM of M13 forward 5′GTAAAACGACGGCCAG 3′ and M13 reverse 5′CAGGAAACAGCTATGAC 3′ primers (synthesized by IDT, BC, Canada), 3.5 mM MgCl_2_ and 2 µl of colony dissolved in 20 µl dH_2_O per reaction. Screening reactions were amplified with an initial denaturation step at 94°C for 3 minutes followed by 30 cycles of 94°C for 30 seconds, 50°C for 30 seconds and 72°C for 45 seconds, ending with 72°C extension step for 7 minutes. The screening amplification reaction produced single products and these were diluted to 200 ng/µl and sequenced at Canadian Centre of DNA Barcoding, Guelph, ON, using M13 forward and reverse primers. Sequences are available at http://www.barcodinglife.org.

### Sequence analysis

COI barcodes for cultivated and uncultivated environmental dinoflagellates used in this study are listed in Tables S1 and S2 respectively, and sequences can be retrieved from the Barcode of Life Database (BOLD) at http://www.boldsystems.org/views/login.php. Cultured sequences 1-332 are listed under DACOI, and all other uncultured sequences under DINOB. Genbank accession numbers GQ501108-GQ502113 and HM236191-HM236201 are also available for all barcodes and listed in Tables S1 and S2. Sequences of cloned products for pseudogene analysis are listed in BOLD within DACL project. All sequences were manually edited using Sequencher™ v4.2 (Gene Codes Corporation, Ann Harbor, USA) and BioEdit™ 7.09 [103]. Nucleotide sequences were translated to the amino acids, aligned and translated back to the nucleotides using BioEdit COI sequences were checked for reading frame interruptions that might indicate the presence of pseudogenes, except where they corresponded to known RNA editing sites in published sequences [78] or had common changes to multiple strains of the same species. To determine the best tree-building method, several algorithms were chosen including maximum likelihood, neighbor-joining using the Kimura-2 distance substitution model (data not shown) and neighbor joining with uncorrected distances using PAUP* 4.0b10 [104]. There was virtually no difference in the topologies produced with any of these methods, so a neighbor joining with uncorrected distance was used in order to compare with pairwise distances between strains which was calculated using PAUP* 4.0b10. The resulting tree or clustergram was visualized by ITOL [105] and Adobe illustrator™ CS2 12.0.0 (San Jose, CA, USA). To calculate cut-off values for a species, only strains identified to species level were used in order to provide an objective comparison of how well COI barcodes corresponded with known species. All unique strains with no matching COI barcode plus *Alexandrium*, *Protoceratium*, *Lingulodinium* were excluded from such calculations, since COI was ineffective at discriminating species in *Alexandrium* or genera in the latter two taxa. *Symbiodinium* was also excluded from initial species and genera level- O.T.U calculations, because species definitions did not apply, this genus being primarily defined by clades. However, once cut-off values were established, these were used to group *Symbiodinium* strains. Out of *Gymnodinium*, only *G. catenatum* and *G. impudicum*, belonging to *Gymnodinium sensu strictu* group, were included for calculations to find species and genera level cut off values, the rest being paraphyletic and therefore unsuitable. Whenever the species category did not match COI barcode O.T.U (such as when more than one named species fell into a single species-O.T.U), the strains were given an O.T.U name that represented all members. The names of strains with only one representative not matching any barcode in this database remained the same. To detect the presence of pseudogenes, the sequences derived from cloned barcodes of a strain were grouped together with that strains directly sequenced product. An average PWD for each strain group was calculated with MEGA software [106] using uncorrected p-distance model including transition and transversion substitutions, homogeneous lineage pattern with uniform site rate. Within group variation was checked manually for unusually high genetic divergence values that might point to presence of a paralogue.

In calculating genus-level O.T.U for environmental barcodes, we categorized each PWD comparison between species and ordered them according to increasing PWD. Each strain was checked and categorized so that every member of a genus-O.T.U group had no more than 1.4% identity to any other member of the same group. Although at the lower end of PWD of known genera, this value also minimized ambiguous identities, where a strain showed equal identity to more than one genus, or showed identity to only some but not all members of a genus-level O.T.U group. In these cases, the corresponding sequences were checked for sequence quality and excluded from analysis if below quality of other sequences. Even with sequence quality parsing, unresolved cases remained. These sequences were removed from their original group and placed in a separate smaller grouping or on their own, for single strains, as shown in Fig. 3 and Fig. S2. Environmental barcodes that did not group with known genera and species were prefixed with EB and a genus number followed by a species number e.g. EB1-1. Genera were labeled according to their groupings in Fig. S2. Some genera were split e.g. *Prorocentrum*, genus 1 was assigned to those barcodes showing closest similarity to *P. levis* and *P. lima* whereas genus 2 was used to denote those barcodes similar to *P. micans, P. minimum* and *P. triestinum*. The only exception were those strains known to belong to Family Kareniaceae, where species in this group were called RS24- if they belonged to the same species as cultured dinoflagellate RS24, and Karenieaceae RS24, if they had genus level identity with that strain.

For Fig. S3. Principal coordinates analysis (PCoA) was performed in GenAlEx 6.2 [107] in order to explore further the relationships between environmental samples and culture collections. This multivariate ordination technique finds the orthogonal axes along which the variation among points described by a distance matrix is greatest. Pairwise distances among all cultured and environmental samples were imported into GenAlEx and squared to produce the matrix required for PCoA, which was performed on covariances and standardized.

## Supporting Information

Figure S1 Neighbor Joining Cluster Analysis of Uncorrected PWD from All Culture Collection COI Barcodes as in Figure 2, with Strain Names.
(1.58 MB TIF)

Figure S2 Neighbor Joining Cluster Analysis of Uncorrected PWD from Culture Collections and Marine Environmental COI Barcodes as in Figure 3, with Strain Names.
(0.75 MB TIF)

Figure S3 Principle Coordinate separation of DNA Barcodes from Cultured and Environmental Dinoflagellates. PCoA using the first three principle coordinates (labeled all-st-1, 2 or 3) of all environmental barcodes and culture collection strains, colour-coded in a similar manner to Fig. 3, except that cultured strains are shown in black. Most cultured strains plus a proportion of environmental clones are in two large cloud points indicating similar variance to each other. The diverse *Symbiodinium* strains (circled in grey) are the only cultured strains are more distant to the majority of cultured dinoflagellates. Most of the pale red barcodes (circled in orange) belong to *Adenoides* sp. from July 2006 together with known *Adenoides* sp. The pale blue barcodes (circled in blue) are an abundant, unidentified genus-level O.T.U. that represented almost the entire diversity of 10 m winter sample from Saanich Inlet. These are the only seasonally related clusters. The small cluster (circled in red) mostly correspond to unidentified groups EB23 and EB24 from Saanich Inlet and are exclusively deep water, from 100–120 m. RS-24 (shown by asterix) is labeled within the Kareniaceae (circled in black). K label indicates position of cultured members of Kareniaceae.
(0.75 MB TIF)

Table S1 Identification of Cultivated and Genbank deposited Dinoflagellate Strains in this Study Using COI barcodes. PWD cut-off of Species-O.T.U is 0.24% or less, Genera 0.T.U is 1.4% or less. Strain Synonyms are indicated in brackets. Identification of COI barcode are explained in the materials and methods. Cross in brackets indicates a strain is over the species cut-off value for its group, explained in main text. Star indicates a strain shows either partial identity with its barcode group or identity to more than one barcode group due to insufficient marker resolution. Misidentified strains are highlighted in italics.
(0.16 MB XLS)

Table S2 Uncultivated Environmental Barcodes in this Study from Dinoflagellate-specific Amplified DNA and from Individual Dinoflagellate Cells. O.T.Us were defined according to PWD comparison cut off values, based on values from known species comparisons (Species is 0.24% or less, genera is 1.4% or less). Naming of COI barcodes are explained in materials and methods. Asterix indicates no image is available.
(0.17 MB XLS)

Table S3 Within group average calculations for seven species to determine presence of paralogues in COI barcodes. Five clones were sequenced per strain, except for CCMP1746 (3 clones) showing that clonal variation in all but one strain was less than 0.2%. Clones of CCMP421 revealed almost 10 times as much diversity compared to that of other dinoflagellates, including another *Symbiodinium* strain.
(0.03 MB XLS)
