# Supplementary figures and images for: Environmental Barcoding Reveals Massive Dinoflagellate Diversity in Marine Environments
Source: PLoS One. 2010 Nov 15;5(11):e13991. doi: 10.1371/journal.pone.0013991 (PMC2981561; doi:10.1371/journal.pone.0013991)

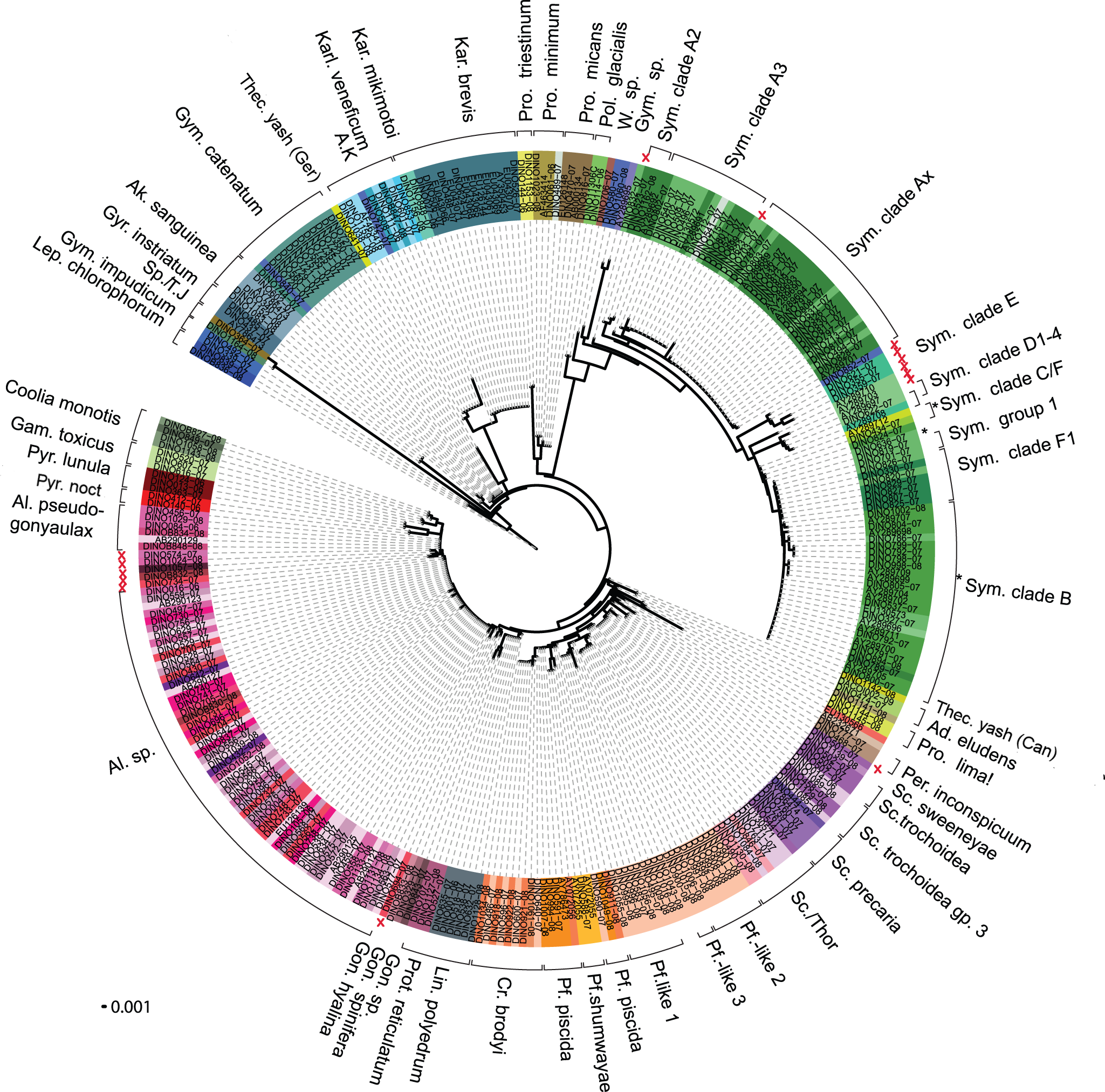

Supplement: Figure S1 — Neighbor Joining Cluster Analysis of Uncorrected PWD from All Culture Collection COI Barcodes as in Figure 2, with Strain Names. (1.58 MB TIF) [file pone.0013991.s001.tif]

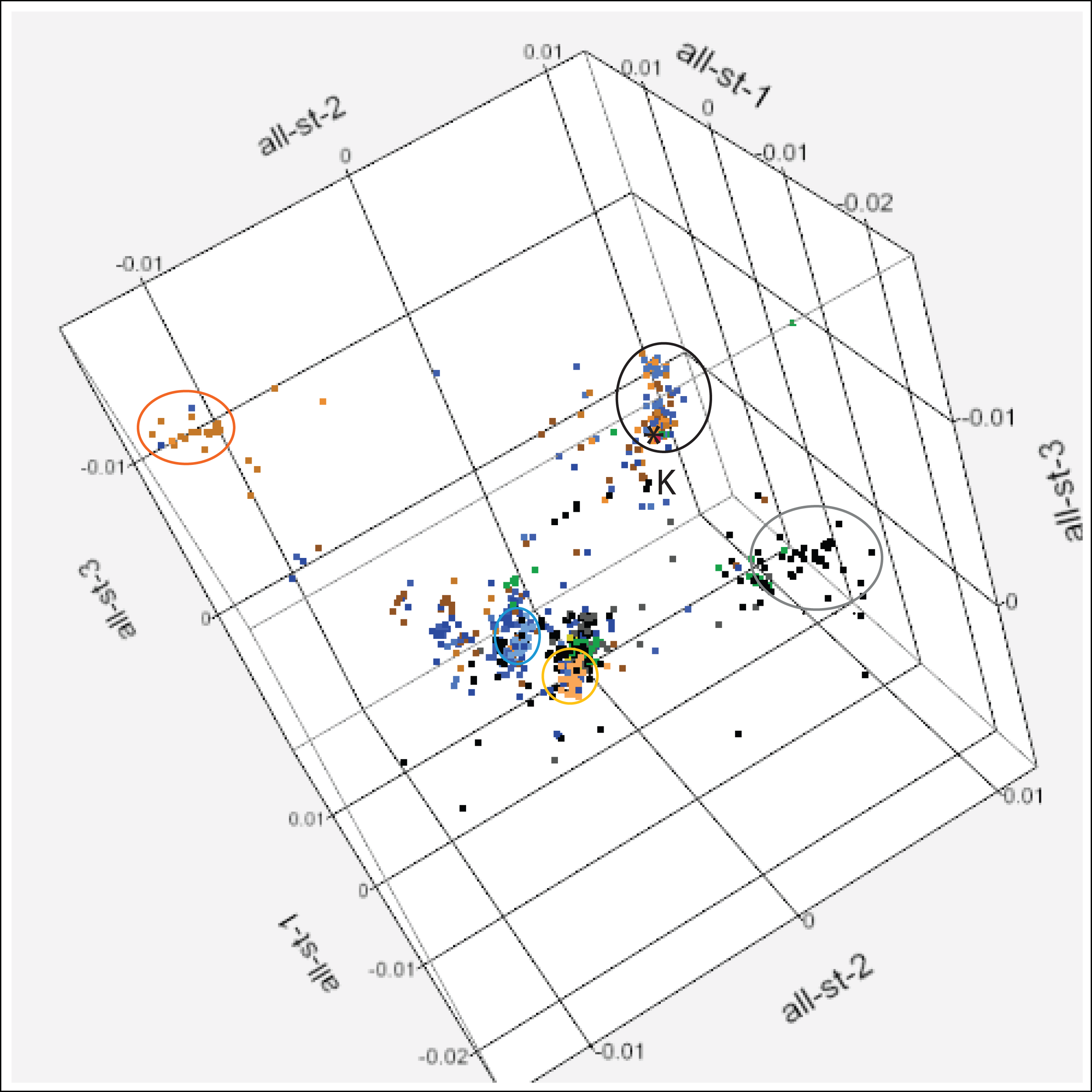

Supplement: Figure S2 — Neighbor Joining Cluster Analysis of Uncorrected PWD from Culture Collections and Marine Environmental COI Barcodes as in Figure 3, with Strain Names. (0.75 MB TIF) [file pone.0013991.s002.tif]
